# PLZF Expression during Colorectal Cancer Development and in Normal Colorectal Mucosa according to Body Size, as Marker of Colorectal Cancer Risk

**DOI:** 10.1155/2013/630869

**Published:** 2013-11-14

**Authors:** Francesco Mariani, Paola Sena, Giulia Magnani, Stefano Mancini, Carla Palumbo, Maurizio Ponz de Leon, Luca Roncucci

**Affiliations:** ^1^Department of Diagnostic, Clinical and Public Health Medicine, University of Modena and Reggio Emilia, 41124 Modena, Italy; ^2^Department of Biomedical, Metabolic and Neural Sciences, University of Modena and Reggio Emilia, 41124 Modena, Italy

## Abstract

Promyelocytic leukemia zinc finger protein (PLZF) is a protein involved in various signaling, growth regulatory, and differentiation pathways, including development/function of some T cells. Here, we aimed at the detection of PLZF during colorectal carcinogenesis, using immunofluorescence, and at the evaluation of the colocalization of PLZF with CD2 and CD56 positive cells (T, **γ** **δ**, NK, and NKT cells), using confocal-microscopy, along colorectal carcinogenesis, since its earliest stages, that is, dysplastic aberrant crypt foci (ACF). Furthermore, we analyzed PLZF in the normal colonic mucosa (NM) according to anthropometric parameters of the subject. NM exhibited strong CD56 fluorescent staining. This infiltration was lost in both ACF and colorectal carcinoma (CRC), while PLZF presence increased from NM to ACF and CRC. Strong association was found between CD56+ colonic mucosa cell infiltration and body mass index. Interestingly, an increased stromal PLZF-reactivity was present in NM of obese subjects. This study shows that overexpression of PLZF and exclusion of NK cells in dysplastic microenvironment are very early events in the stepwise sequence leading to CRC and that lower levels of CD56+ cells in NM, together with increased levels of PLZF+ cells, can be a reflection of colon cancer risk due to obesity.

## 1. Introduction

The zinc finger and BTB domain containing 16 (*Zbtb16*) gene, described in humans since 1993, encodes for promyelocytic leukemia zinc finger protein (PLZF), a sequence-specific DNA-binding protein which bears a conserved N-terminal BTB/POZ repression and multimerization domain, a second region (RD2) responsible for transcriptional repression, and a C-terminal with nine C2H2 Kruppel-type zinc finger motifs that create the DNA-binding domain [1, 2]. Similar to other members of this family (approximately 60 human BTB/POZ-zinc fingers have been described, including HIC-1, BCL-6, Kaiso, FAZF, and LRF), PLZF has generally transcriptional repressor activity; however, PLZF can also activate transcription. The determinants of activator versus repressor function have not been defined. The BTB/POZ domain is also strongly implicated in the control of transcriptional activation or repression through local chromatin modification and remodeling. PLZF interacts directly with nuclear proteins such as mSin3a, SMRT, and NCoR via conserved residues in its BTB/POZ domain and, in turn, interacts with histone deacetylases (HDAC1) [3, 4].

Earlier studies highlighted the role of PLZF in regulating differentiation. These reports confined the expression of PLZF to stem cells and early progenitor cells, suggesting that PLZF is essential for preserving pluripotency and the ability to self-renew [5–7]. PLZF is expressed at high levels in undifferentiated, multipotential hematopoietic progenitor cells, and this expression declined when those cells were committed to a specific hematopoietic lineage and started to differentiate. By contrast, overexpression of PLZF turns off the production of mature cells, regulating the balance between proliferation and differentiation [8, 9].

It is now known that PLZF is expressed in many cells of the body, and several lines of evidence indicate that PLZF participates in various signaling, growth regulatory, and differentiation pathways, including hematopoiesis, adipogenesis, hippocampal neurogenesis, osteoclastogenesis, and muscle differentiation, and it is involved in diverse functions, such as homeostasis, neoplasia, and apoptosis [10, 11].

Recent data have shown that PLZF is expressed only in specific populations of T cells. PLZF was required for acquisition of the innate-like properties of these cells, such as rapid cytokine production, ability to produce simultaneously Th1 and Th2 cytokines, and exhibiting an activated phenotype. Several laboratories reported that PLZF plays critical roles in the development or function of some cells such as natural killer cells (NK), natural killer T cells (NKT), gammadelta T cells (*γδ* T cells), and memory CD8 T cells [12–15]. 

NK cells are a subset of lymphocytes which represent 5% to 20% of peripheral blood mononuclear cells, and they are defined phenotypically by their expression of CD56 antigen and lack of expression of CD3 one. NK cells are a component of the innate immune system, and they have the ability to both lyse target cells and to provide an early source of immunoregulatory cytokines. NK cells play an important role in the defence against viral infections as well as in tumor surveillance. At present, it is accepted that NK cells are a subset of lymphocytes with an important role in the early response to tumors [16, 17].

PLZF has been also identified as a key transcription factor involved in the development of natural killer T (NKT) cells. NKT cells are a subset of T cells coexpressing the T cell receptor (TCR)/CD3 complex along with surface receptors typical of NK cells. NKT cells are known for their ability to produce a massive burst of cytokines early in an immune response. NKT cells are multifunctional, they can enhance microbial immunity and tumor rejection, suppress autoimmune disease, and promote tolerance [18].

Gammadelta T lymphocytes (*γδ* T cells) are a small subset of T cells that possess a distinct T-cell receptor (TCR) on their surface. They protect epithelial barriers via monitoring and appropriate destruction of dysregulated or transformed epithelial cells. PLZF can be induced in polyclonal, immature but not mature gammadelta T lymphocytes, governing acquisition of “innate” properties [19]. 

Some studies reported that PLZF affects body weight, adiposity, lipid profile, insulin sensitivity of skeletal muscle, and related gene expression. Recent lines of evidence clearly indicate that major pathways relevant for metabolic syndrome (a condition characterized by clustering of insulin resistance, dyslipidaemia, obesity, hypertension, and procoagulant state) converge to this factor [20].

PLZF functions as a transcriptional regulator for cell cycle progression by binding to the promoter of target genes, such as those for cyclin A and the interleukin-3 receptor *α* subunit. PLZF causes growth suppression and cell cycle arrest through its ability to repress expression of a number of growth promoting genes and protooncogenes. The loss of PLZF has been related to increased proliferation, invasiveness and motility, and resistance to apoptosis in different cancer cell types. PLZF is considered a tumor suppressor gene in various cell types and tissues. However, little is known about its putative role in solid tumors [21, 22].

In this study, we aimed at the detection, morphological localization, and quantification of PLZF in human colonic mucosa and along colorectal carcinogenesis. We looked for a colocalization with CD2, a glycoprotein that is expressed on T, *γδ*-T, and natural killer (NK) cells [23], and with CD56, a markers of NK and NKT cells, since the stage of dysplastic aberrant crypt foci, (ACF) [24, 25], referred to also as microadenomas, microscopic lesions considered the earliest events in the multistep process of colorectal carcinogenesis. Here, we report the substantial expansion of PLZF-expressing cells during colorectal cancer progression, and this is regardless of the simultaneous decrease in the NK/NKT cells content. Furthermore, we analyzed the presence of this protein in the colonic mucosa of subjects according to body mass index, in order to assess a potential involvement of this protein in the definition of colorectal cancer risk.

## 2. Materials and Methods

### 2.1. Study Population

Waist circumference and body mass index (BMI) based on weight and height of patients were measured from patients who underwent colonoscopy for various reasons. Seven subjects with anthropometric measures above the cut-off levels for obesity (BMI ≥ 25 Kg/m^2^ and waist circumference ≥ 88 cm for women and ≥102 cm for men) and 7 subjects with anthropometric measures under the cut-off levels of obesity (BMI < 25 Kg/m^2^ and waist circumference < 88 cm for women and <102 cm for men) were enrolled in the study. One sample of normal colorectal mucosa (NM) was collected from each of the 14 patients during colonoscopy, and it was frozen at −80°C. All patients had normal colonoscopy. Demographic and anthropometric data of these patients are shown in Table 1. Furthermore, at least one aberrant crypt focus (ACF) was removed from each of 10 patients after operation for colorectal cancer on surgical specimens, after staining of the mucosa with a 0.1% methylene-blue solution in saline, and observation under a dissecting microscope [24, 25], and it was frozen at −80°C. Finally, a sample of colorectal cancer (CRC) was collected from 10 patients whom where operated on for colon or rectal cancer and frozen at −80°C, as described above.

All patients enrolled in this study, who underwent colonoscopy or surgical resection for colorectal cancer at the University Hospital of Modena, were asked to give an informed written consent to the study protocol, which was approved by the Comitato Etico Provinciale di Modena.

### 2.2. Evaluation of Immunofluorescence by Confocal Microscopy

The samples of NM, ACF, and CRC were fixed in 4% paraformaldehyde in PBS, cryoprotected in 15% sucrose in PBS, and frozen in isopentane cooled in liquid nitrogen. Horizontal cryosections of the samples were cut (10 *μ*m thick), and haematoxylin and eosin staining was performed on sections to control tissue integrity and histology. After a treatment with 3% BSA in PBS for 30 min at room temperature, the cryostatic sections were incubated with the primary antibodies: rabbit anti-zbtb16 (Sigma), mouse anti-CD2, and mouse anti-CD56 (Dako), diluted 1 : 25 in PBS containing 3% BSA for 1 h at room temperature.

After washing in PBS, the samples were incubated for 1 h at room temperature with the secondary antibodies diluted 1 : 20 in PBS containing 3% BSA (sheep anti-mouse FITC conjugated and goat anti-rabbit TRITC conjugated (SIGMA)). After washing in PBS and in H_2_O, the samples were counterstained with 1 *μ*g/mL DAPI in H_2_O and then mounted with antifading medium (0.21 M DABCO and 90% glycerol in 0.02 M Tris, pH 8.0). Negative control samples were not incubated with the primary antibody. The confocal imaging was performed on a Leica TCS SP2 AOBS confocal laser scanning microscope.

Excitation and detection of the samples were carried out in sequential mode to avoid overlapping of signals. Sections were scanned with laser intensity, confocal aperture, gain and black-level setting kept constant for all samples. Optical sections were obtained at increments of 0.3 *μ*m in the *z*-axis and were digitized with a scanning mode format of 512 × 512 or 1024 × 1024 pixels and 256 grey levels. The confocal serial sections were processed with the Leica LCS software to obtain three-dimensional projections. Image rendering was performed by adobe Photoshop software. The original green fluorescent confocal images were converted to grey-scale, and median filtering was performed. An intensity value ranging from 0 (black) to 255 (white) was assigned to each pixel.

Background fluorescence was subtracted, and immunofluorescence intensity (IF) was calculated as the average for each selected area. The fluorescence intensity at the selected areas, linearly correlated with the number of pixels, was quantitatively analysed using the standard imaging analysis software of an NIS-Elements system. To each sample was assigned a code number, and the score, referred to as immunofluorescence intensity score (IFIS), was determined by an observer who was blind to tissue groups during analysis [26, 27].

### 2.3. Colocalization Analysis

To examine the cellular localization of PLZF within CD2+ and CD56+ cells, multiple immunofluorescence staining of rabbit anti-PLZF antibody, (Sigma) with mouse anti-CD2 and mouse anti-CD56 (Dako), were applied according to our previously published method.

The samples, processed for multiple fluorescence (DAPI, FITC, and Cy3), were sequentially excited with the 405 nm/25 mW lines of a blue diode laser, the 488 nm/20 mW lines of the Argon laser, and the 543 nm/1.2 mW lines of a HeNe laser.

Optical sections were obtained at increments of 0.3 *μ*m in the *z*-axis and were digitized with a scanning mode format of 512 × 512 or 1024 × 1024 pixels and 256 grey levels. For colocalization analyses, the different channel images were acquired independently, and photomultiplier gain for each channel was adjusted to minimize background noise and saturated pixels. Once acquired, images were not modified further. 

The degree of colocalization between red (Cy3) and green signals (FITC) was calculated based on the integrated density of each signal independently for each different sections. Manders' coefficient was calculated with ImageJ using the JACoP tool [28]. Manders' coefficient varies from 0 to 1, corresponding to nonoverlapping images and 100% colocalization between the two images, respectively. The intensity of fluorescence for each marker was multiplied for the corresponding Manders' coefficient, in order to obtain the normalized colocalization levels.

### 2.4. Statistical Analysis

All quantitative data for NM, ACF, and CRC are reported as mean ± SE. The difference in average expression, in the different groups of colorectal lesions, was tested for statistical significance using Kruskal-Wallis analysis, followed by Student-Newman-Keuls tests. The value of *P* < 0.05 was chosen to indicate a significant difference.

## 3. Results

### 3.1. Fluorescence Analysis of CD2+ and CD56+ Cell Infiltration during Colorectal Carcinogenesis

**Figure 1:**
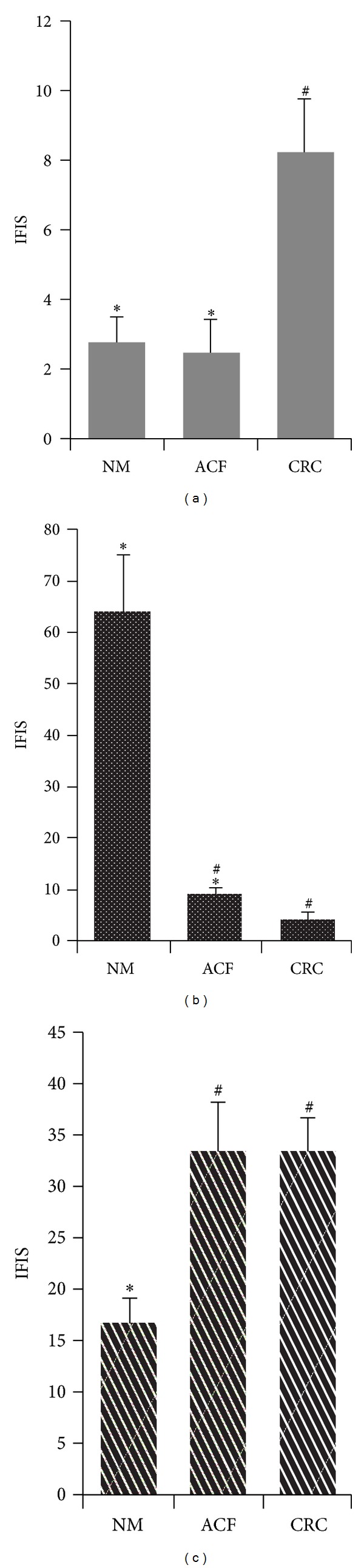
CD2, CD56, and PLZF fluorescence levels along colorectal carcinogenesis. Fluorescence levels of CD2 (a), CD56 (b), and PLZF (c) immunostaining in normal colorectal mucosa (NM), aberrant crypt foci (ACF), and colorectal carcinoma (CRC), expressed by immunofluorescence intensity score (IFIS). ^#^ *P* < 0.05 versus NM; *P < 0.05 versus CRC.

**Figure 2:**
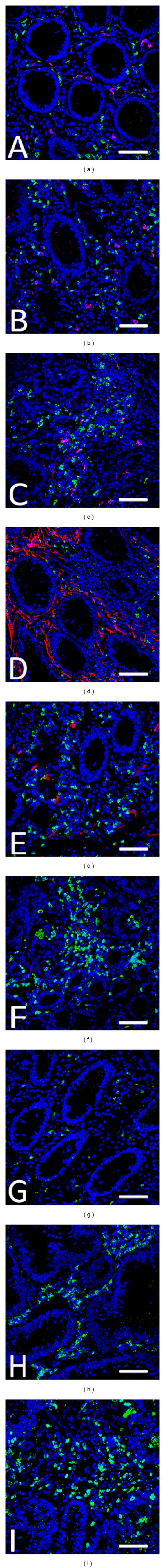
Confocal immunofluorescence staining of colorectal carcinogenesis. Examples of confocal analysis of cryosections of normal colorectal mucosa (NM), aberrant crypt foci (ACF), and colorectal carcinoma (CRC), labelled by DAPI (blue), PLZF (green), CD2 (magenta), and CD56 (red). (a)–(c): PLZF with CD2 in NM (a), ACF (b), and CRC (c). (d)–(f): PLZF and CD56 in NM (d), ACF (e), and CRC (f). (g)–(i): immunostaining of PLZF alone in NM (g), ACF (h), and CRC (i). Scale bar = 80 *μ*m.

We performed an immunofluorescence analysis with anti-CD2 antibody on normal colonic mucosa, ACF, and colorectal cancer. This adhesion protein can discriminate NK cell, T lymphocytes, and gammadelta T cells. The number of CD2+ cells was generally low in normal colonic mucosa (IFIS: 2.7 ± 0.7). This fact was also evident in ACF (IFIS: 2.6 ± 0.9), despite a well-documented inflammatory microenvironment previously reported in these lesions. On the other hand, we observed an increased level of CD2-associated fluorescence in colorectal carcinomas (IFIS: 8.2 ± 1.5), and this gain was statistically significant with respect to NM and ACF (Figures 1(a), 2(a), 2(b), and 2(c)).

By contrast, we observed a very strong CD56 fluorescence staining in normal colorectal mucosa (IFIS: 64.3 ± 11.1). The presence of CD56+ cells decreased dramatically in ACF (IFIS: 9.4 ± 1.4). Finally, CD56 fluorescent staining was further reduced in colorectal carcinomas until almost undetectable (IFIS: 4.2 ± 1.4) (Figures 1(b), 2(d), 2(e), and 2(f)).

### 3.2. Morphological Analysis of PLZF+ Cell Infiltration and Spatial Relationship with CD2 and CD56

In samples of colorectal mucosa, the anti-PLZF antibody was used firstly to define the spatial and cellular expression of this protein. Examples of PLZF staining are shown in Figures 2(g), 2(h), and 2(i). Our experiments consistently showed that PLZF protein is expressed only in the stromal compartment of all samples of colonic mucosa and lesions, with no sign of epithelial staining. 

Although there were a few PLZF-positive cells in the stroma of normal colonic mucosa (IFIS: 16.6 ± 2.4), their number increased sharply in the neoplastic milieux. Indeed, we observed a significant increase of the PLZF fluorescence signals in ACF (IFIS: 33.5 ± 4.8). The number of PLZF+ infiltrating cells remained high in the stroma surrounding colorectal cancer cells (IFIS: 33.7 ± 3.1) (Figures 1(c) and 2(g)–2(i)).

We also performed double fluorescent staining of PLZF with CD2 and of PLZF with CD56. The colocalization levels were measured with the Manders' coefficient values (data not shown). As shown also in Figure 2, PLZF protein colocalized with none of the markers, and the proteins were almost mutually exclusive.

### 3.3. CD2+, CD56+, and PLZF+ Cell Infiltration in Normal Colonic Mucosa according to Anthropometric Parameters of Subjects with Normal Colonoscopy

**Figure 3:**
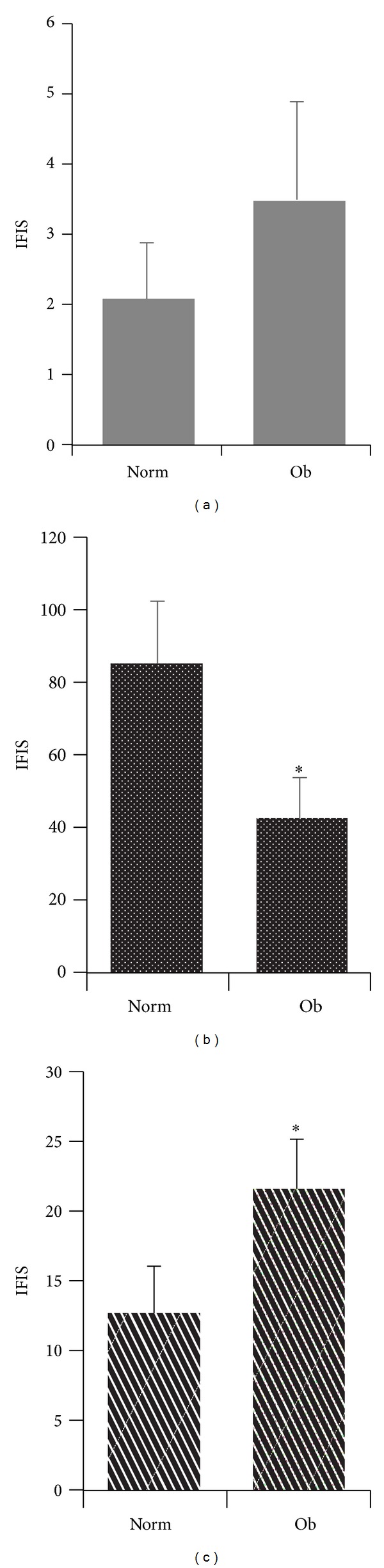
CD2, CD56, and PLZF fluorescence levels in normal colorectal mucosa of subjects with normal colonoscopy. Fluorescence levels of CD2 (a), CD56 (b), and PLZF (c) immunostaining in normal colorectal mucosa of subjects with normal weight (Norm: BMI < 25 Kg/m^2^ and waist circumference < 88 cm for women and <102 cm for men) and obese subjects (Ob: BMI ≥ 25 Kg/m^2^ and waist circumference ≥ 88 cm for women and ≥102 cm for men) expressed by immunofluorescence intensity score (IFIS). *P < 0.05 versus Norm.

**Figure 4:**
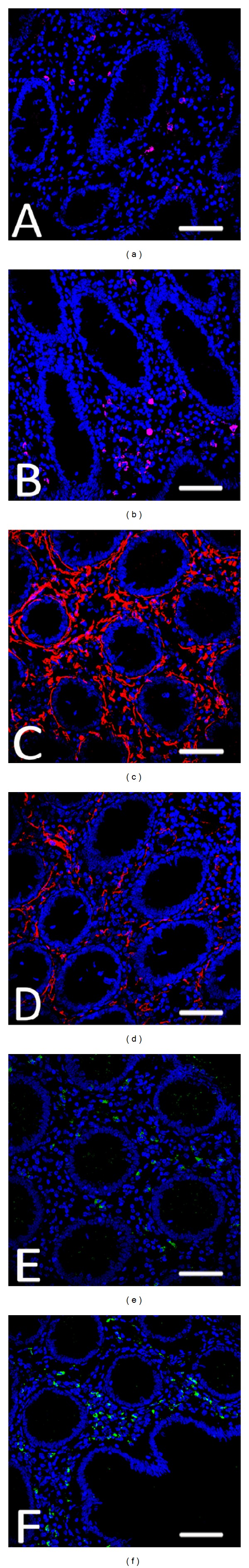
Immunofluorescence staining of normal colorectal mucosa. Examples of immunofluorescence of normal colorectal mucosa labelled by DAPI (blue), CD2 (magenta, (a)-(b)), CD56 (red, (c)-(d)), and PLZF (green, (e)-(f)) in subjects with normal weight (BMI < 25 Kg/m^2^ and waist circumference < 88 cm for women and <102 cm for men; (a),(c), and (e)) and obese subjects (BMI > 25 Kg/m^2^ and waist circumference ≥ 88 cm for women and ≥102 cm for men; (b), (d), and (f)). Scale bar = 80 *μ*m.

We divided patients who underwent colonoscopy according to their anthropometric parameters (body mass index and waist circumference) in order to relate the inflammatory content of normal colonic mucosa, as assessed by the markers used (mainly NK and NKT cells), with parameters indicative of increased colorectal cancer risk. All three markers showed differences in their colonic content on the basis of body composition of the subject. Particularly, there was a substantial decrease of CD56+ cells in the normal colonic mucosa of obese subjects (IFIS: 84.8 ± 17.1 in normal people versus IFIS: 42.9 ± 10.5 in obese people). These subjects also showed increased levels of CD2+ cells compared to the levels in the normal colonic mucosa of normal weight subjects, although the difference was not significant (IFIS: 2.1 ± 0.7 in normal weight subjects versus IFIS: 3.5 ± 1.4 in obese subjects). Also PLZF showed higher expression in normal colonic mucosa of obese subjects, and the difference was significant (IFIS: 12.7 ± 3.3 in normal weight subjects versus IFIS: 21.5 ± 3.6 in obese subjects) (Figures 3 and 4).

## 4. Discussion

In this study, presence, tissue localization, and amount of PLZF in normal colorectal mucosa were evaluated together with presence and amount of CD2- and CD56-positive cells. Furthermore, we also investigated the fluorescence levels of these proteins in early premalignant lesions of the colonic mucosa, that is, dysplastic aberrant crypt foci (ACF), and in colorectal cancer microenvironment.

CD2 is a cell-surface glycoprotein expressed only on T cells, natural killer (NK) cells, and dendritic cells. It plays an important role in regulating cell responses; indeed, it can initiate innate and adaptive immune responses. Here, we found a small amount of CD2+ cells in normal colorectal mucosa. An increase in CD2+ cells was found only in colorectal cancer samples, while the amount of CD2+ cells remained low in ACF.

Cell surface expression of CD56 phenotypically defines NK cells and NKT cells. Normal colorectal mucosa exhibited a strong fluorescent staining of CD56-positive cells. This infiltration was lost early, since the earliest appearance of dysplastic epithelium, as in ACF. Moreover, carcinomatous tissue was poorly infiltrated by CD56+ cells.

Classically, PLZF has been considered a master regulator of stem cells and early progenitor cells maintenance. However, several recent evidences point out that PLZF is expressed in many cells and tissue of the body such as myocytes, skeletal muscles, and renal tubules. A recent paper highlights the presence of a specific PLZF isoform in intestinal epithelial cells [29]. Here, we found that the classical PLZF isoform is detectable by immunofluorescence techniques only in the stromal compartment of colorectal mucosa. The presence of PLZF+ cells increased from normal colorectal mucosa to ACF, and it remained high also in colon cancer. 

This study is in line with the hypothesis that the immune response is impaired since the earliest phases of colorectal cancer development. Indeed, CD2 has a role in both T-cell adhesion and activation processes [30], and it is involved in immune surveillance. We observed an increase in the levels of CD2 only in cancer tissue, when the immune response is almost unable to fight the tumor. Thus, this is evidence that colorectal cancer-driven modifications in the immune response appear since the stage of ACF.

Also, NK cells function well as effector cells against tumor target cells. If NK cells are not capable of infiltrating solid tumors and preneoplastic lesions, they cannot kill cancer cells when they appear. Despite others studies that reported that there is a low NK-cell numbers in CRC [31], this study shows, for the first time, that the exclusion of NK cells from the stroma surrounding the tumor is a very early event in the stepwise sequence leading to cancer development.

Several works demonstrated that phenotype and function of lymphocytes expressing PLZF are altered [32]. It has also been reported that PLZF-deficient cells have a depletion of the progenitor pool, while they generate an increased amount of mature progeny. Some authors hypothesized that overexpression of PLZF can decrease the production of mature cells in favor of a maintenance of undifferentiated progenitors [33]. Our study fits this hypothesis, since PLZF expression increases from early dysplastic lesions, envisaging a central role for PLZF in the mechanism leading to immune-escape. Indeed, an overexpression of PLZF in dysplastic microenvironment may lead to decreased number of fully mature effector cells. Thus, a uneffective cytotoxic reaction can be permissive toward a neoplastic growth. 

The initial mechanism of cancer immunosurveillance is probably nonspecific, but NK cells seem to play a key role at this stage. Although the importance of immune surveillance against cancer, in particular that provided by NK cells, has been indicated in several papers [16, 17], few studies have investigated the levels of NK-cell in individuals who have an increased cancer risk. 

There is now strong evidence indicating obesity as a risk factor for various cancers, particularly colorectal, breast, uterus, prostate, and pancreatic cancer. Obesity is associated with a proinflammatory state, and it may be an immune-impairing condition too. It has been reported that obesity has complex and probably suppressive effects on NK lymphocytes [34]. We found a strong association between CD56+ cell infiltration of the normal colonic mucosa and body mass, and the normal colonic mucosa of obese people has decreased levels of CD56-immunoreactivity. We also observed an increased stromal PLZF-reactivity in the normal colonic mucosa with obesity, prefiguring, as reported by several studies, a relationship between this protein and the main energetic and adipogenetic pathways, although the mechanisms of this association are still unknown. 

In conclusion, it has been proposed that there is an association between natural immunological defence and the occurrence of common cancers. Our results seem to suggest that lower levels of CD56+ cells in normal colonic mucosa, together with increased levels of PLZF+ cells, can mean the expression of an inhibitory mechanism of colorectal cancer development. Furthermore, the variation of these markers in normal colonic mucosa taken during colonoscopy may be associated with colon cancer risk through alterations induced by obesity.

## Figures and Tables

**Table 1:** Demographic and anthropometric data of subjects with normal colonoscopy.

Patient number	Age	Sex	BMI	Waist circumference
1	79	M	25.9	103
2	79	F	23	85
3	52	F	24.2	81
4	63	F	21.25	85
5	46	M	31	111
6	41	M	32.5	117
7	46	F	19.9	75
8	68	F	28.66	107
9	65	F	28.84	99
10	56	M	24.3	98
11	64	M	24.16	92
12	73	M	29	109
13	68	M	34.4	116
14	55	M	24.30	97
